# *Latilactobacillus curvatus*: A Candidate Probiotic with Excellent Fermentation Properties and Health Benefits

**DOI:** 10.3390/foods9101366

**Published:** 2020-09-25

**Authors:** Ying Chen, Leilei Yu, Nanzhen Qiao, Yue Xiao, Fengwei Tian, Jianxin Zhao, Hao Zhang, Wei Chen, Qixiao Zhai

**Affiliations:** 1 State Key Laboratory of Food Science and Technology, Jiangnan University, Wuxi 214122, China; edyulei@126.com (L.Y.); cyingjiangnan@163.com (Y.C.); nanzhen.qiao@gmail.com (N.Q.); xiaoyue_jiangnan@163.com (Y.X.); fwtian@jiangnan.edu.cn (F.T.); zhaojianxin@jiangnan.edu.cn (J.Z.); zhanghao61@jiangnan.edu.cn (H.Z.); chenwei66@jiangnan.edu.cn (W.C.); 2 School of Food Science and Technology, Jiangnan University, Wuxi 214122, China; 3 International Joint Research Laboratory for Probiotics, Jiangnan University, Wuxi 214122, China; 4 (Yangzhou) Institute of Food Biotechnology, Jiangnan University, Yangzhou 225004, China; 5 National Engineering Research Center for Functional Food, Jiangnan University, Wuxi 214122, China; 6 Beijing Innovation Centre of Food Nutrition and Human Health, Beijing Technology and Business University, Beijing 100048, China

**Keywords:** *Latilactobacillus curvatus*, probiotic candidate, fermentation properties, beneficial function

## Abstract

*Latilactobacillus curvatus* is a candidate probiotic that has been included in the list of recommended biological agents for certification by the European Food Safety Authority. According to the published genomic information, *L. curvatus* has several genes that encode metabolic pathways of carbohydrate utilization. In addition, there are some differences in cell surface complex related genes of *L. curvatus* from different sources. *L. curvatus* also has several genes that encode bacteriocin production, which can produce Curvacin A and Sakacin P. Due to its ability to produce bacteriocin, it is often used as a bioprotective agent in fermented meat products, to inhibit the growth of a variety of pathogenic and spoilage bacteria. *L. curvatus* exerts some probiotic effects, such as mediating the production of IL-10 by dendritic cells through NF-κB and extracellular regulated protein kinases (ERK) signals to relieve colitis in mice. This review is the first summary of the genomic and biological characteristics of *L. curvatus*. Our knowledge on its role in the food industry and human health is also discussed, with the aim of providing a theoretical basis for the development of applications of *L. curvatus*.

## 1. Introduction

*Latilactobacillus curvatus* is a candidate probiotic that has attracted much attention due to its excellent fermentation properties and health benefits. In 2012, it was listed in the “Catalogue of Microorganisms with Technical Necessity in Fermented Food” of the Bulletin of the International Dairy Federation [1] and was included as a recommended biological agent in the European Food Safety Administration Qualification Certification list in 2013 [2]. *L. curvatus* was approved by the Chinese government as a new food raw material for processing meat, dairy and fish products in 2019. The rapid development of genomic technologies will facilitate greater progress in research on *L. curvatus.*

*L. curvatus* is a member of the genus *Latilactobacillus* (phylum *Firmicutes*, class *Bacilli*). On agar plates, *L. curvatus* colonies appear milky white (diameter of 1–2 mm) [3], opaque and convex, with a neat and moist edge. As for the cellular morphology, members of this species present as curved, bean-shaped rods with rounded ends (0.7–0.9 × 1–2 μm) [3], occurring in pairs or short chains. Closed rings or horseshoes often form, usually comprising four cells [3]. *L. curvatus* is one of the major bacterial species associated with poultry products and fermented meat products [4,5] and thus, is often isolated from fermented meat products. This species has also been isolated from dairy products (milk and cheese) [6]; fermented plant products, such as kimchi [7,8,9] and sourdough [10] and other plant-derived materials, such as honey [11]. Accordingly, *L. curvatus* has been identified in the feces or gut of many animal species that feed on plants or cereals, including snails [12], chickens [13], and humans [14]. 

**Figure 1:**
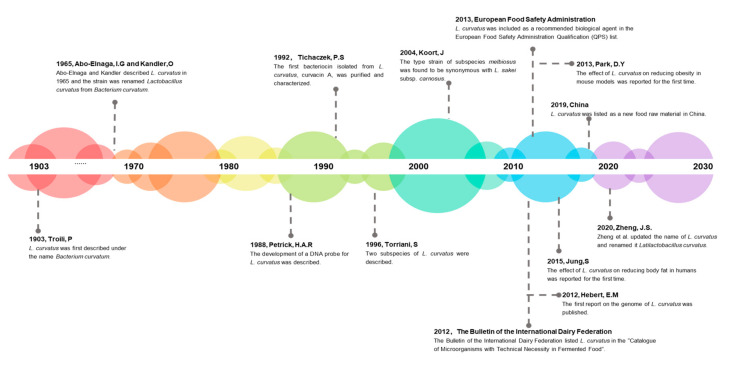
Timeline of selected key findings and technical advances related to *Latilactobacillus curvatus*.

*L. curvatus* was first described under the name *Bacterium curvatum* by Troili in 1903 [3]. Additionally, in 1965, Abo-Elnaga and Kandler described *L. curvatus* and the species was renamed *Lactobacillus curvatus* from *Bacterium curvatum*. [15,16]. In 2020, the name of *L. curvatus* was updated again, and Zheng et al. [17] renamed it *Latilactobacillus curvatus.* (Figure 1) Phylogenetically, *L. curvatus* is closely related to *Latilactobacillus sakei* [18]. The two strains have relatively high DNA sequence homology, and their only differences lie in the hydrolysis of arginine and the fermentation of maltose [19]. Due to their similarity, differentiation between the two species is difficult without any molecular technique and often arbitrary. Thus, Petrick et al. developed a specific DNA probe for *L. curvatus* in 1988 [20]. *L. curvatus* is unique due to its bacteriocinogenic activity. It is a producer of class II bacteriocins such as Curvacin A and Sakacin P which can inhibit pathogenic bacteria such as *Listeria monocytogenes* and *Staphylococcus aureus* [21]. Curvacin A produced by *L. curvatus* LTH1174 was the first bacteriocin to be purified and characterized by Tichaczed et al. in 1992 [22]. It is a class II bacteriocin with inhibitory effects on various pathogenic bacteria. In 1996, two subspecies, *L. curvatus* subsp. *curvatus* and *L. cuwatus* subsp. *melibiosus*, were discovered by Torriani et al. [23]. In 2004, molecular studies by Koort et al. [24] revealed that the type strain of *L. cuwatus* subsp. *melibiosus* was synonymous with *L. sakei* subsp. carnosus. With the continuous development of genome sequencing methods, Hebert et al. [25] sequenced *L. curvatus* for the first time in 2012. *L. curvatus* CRL705 was the first *L. curvatus* strain sequenced. This strain, which was isolated from an Argentinean artisanal fermented sausage, is known as a producer of the two-component bacteriocin lactocin 705 and lactocin AL705 [25]. 

By 2020, the genome sequences of 24 strains of *L. curvatus* had been published in the National Center for Biotechnology Information (NCBI) Assembly database. Prior to this, little genomic data were available for *L. curvatus*, due to a lack of large-scale genetic analyses of this species in different geographical regions. The existing genomic data show that *L. curvatus* has a variety of genes related to multiple carbohydrate metabolic pathways, such as those for glucose and trehalose metabolism [26]. In addition, these data indicate that *L. curvatus* has multifarious genes related to bacteriocin production [27]. *L. curvatus* is often used as a biological protective agent in fermented meat products because of its excellent bacteriocin production ability, which can inhibit the growth of spoilage bacteria [21]. What’s more, the bacteriocin produced by this strain can be sprayed onto polyethylene film to produce active food packaging [28]. Besides bacteriocins, organic acids produced by *L. curvatus* metabolism can reduce the pH of meat product fermentation systems and thus reduce the nitrite content in meat products [29]. The ability of *L. curvatus* to hydrolyze fatty acids also enables these bacteria to impart desirable flavors to meat products [30].

In addition to its application in the food industry, recent studies have shown that *L. curvatus* is beneficial to human health. This strain can effectively reduce the effect of dextran sodium sulfate (DSS)-induced colitis in mice [31]. It can also relieve obesity and hyperlipidemia [32], but it was more effective when mixed with *L. plantarum* [33,34]. A recent study showed that *L. curvatus* can also effectively prevent muscle atrophy induced by dexamethasone, and this was the first study to report on the inhibiting effects of lactic acid bacteria (LAB) on muscle atrophy [35]. In this review, we will focus on *L. curvatus*, discuss its genomic characteristics and physiological and biochemical properties, and introduce its applications in the food industry and human health. This will provide a reference for further studies on *L. curvatus*. 

## 2. Genomic Characteristics of *Latilactobacillus curvatus*

With the continuous development of genome sequencing methods, complete genome sequences have been obtained for different LAB isolated from meat products [25,36]. In 2012, Hebert et al. [25] sequenced *L. curvatus* CRL705, a strain used as a starter culture for sausage fermentation. This was the first *L. curvatus* strain to be sequenced. Subsequently, other *L. curvatus* strains were also sequenced, such as *L. curvatus* FBA2 isolated from fermented vegetables [8], *L. curvatus* WiKim38 isolated from Kimchi [37], *L. curvatus* FLEC03 isolated from beef [38] and *L. curvatus* NFH-Km12 isolated from traditional Japanese fermented fish [39] (Table 1).

The genomes of *L. curvatus* strains range from 1.804 Mb for *L. curvatus* RI198 to 2.132 Mb for *L. curvatus* TMW 1.624. Their genome sizes follow a normal distribution, with an average size of 1.946 Mb. The average G + C content is 41.93%, ranging from 41.63% for *L. curvatus* TMW 1.624 to 42.1% for *L. curvatus* FBA2. The average number of predicted coding sequences (CDS) per genome is 1915, ranging from 1711 for *L. curvatus* FBA2 to 2148 for *L. curvatus* TMW 1.624. Furthermore, some strains carry up to two plasmids. These general genomic characteristics of *L. curvatus* highlight some genetic variation among strains.

Teran et al. [26] conducted a comparative analysis of 13 published genomes (*L. curvatus* NRIC0822, Wikim 38, FBA2, DSM20019, Wikim 52, CRL705, RI-193, RI-198, RI-124, KG6, FLEC03, RI-406, MRS6) and found that the *L. curvatus* core genome contains 6742 sites of single nucleotide polymorphism, which can be used to distinguish two major lineages. Lineage one is represented by the newly sequenced strain, *L. curvatus* FLEC03, and lineage two contains two branches, with branch 2A represented by the type-strain *L. curvatus* DSM20019 and branch 2B represented by *L. curvatus* KG6. Cluster analysis of the core and accessory genomes of the above strains showed that the strains from the 2B branch recently evolved from lineage two and obtained the functional characteristics of lineage one, and that branch 2B shares a separate source with lineage one. Furthermore, strains of the 2A branch were isolated from Asian foods (sushi and kimchi). Therefore, the patterns of the accessory genomes of *L. curvatus* indicate that some features affecting environmental adaptability have only recently been acquired. However, eight of the 13 strains of *L. curvatus* were isolated from fresh or fermented meat, indicating that the isolation sources of these strains are relatively similar, and the number of strains is small. Therefore, it is necessary to compare and analyze more strains from other sources (e.g., gastrointestinal tract, vegetables, silage) to further elucidate the evolutionary model of *L. curvatus*.

Teran et al. [26] studied 13 strains of *L. curvatus* with published genomic information and found that three strains in lineage one had eight putative cell-surface complexes groups, two of which were shared with strains RI-124, RI-198, and RI-193 from branch B of lineage two. However, these gene clusters are absent in the branch A of lineage two which are non-meat strains, suggesting that they might be habitat-specific properties. 

Eisenbach et al. [27] analyzed the gene clusters of 10 strains of *L. curvatus* and found that eight strains contained genes related to bacteriocin production which may encode proteins homologous to Sakacin Q. In addition, the genomes of two of the eight strains were found to contain two bacteriocin gene clusters. In addition to sakacin Q, they encode a putative functional type-A lantibiotic. Furthermore, the genome of *L. curvatus* TMW 1.624 contains four putative bacteriocin gene clusters with genes related to sakacin Q, sakacin Tα, enterocin NKR-5-3a, and a class II lanthipeptide. Based on these data, the genes related to bacteriocin production in *L. curvatus* seem to have little relationship with the source of the strain.

Eisenbach et al. [27] identified the gene cluster of *L. curvatus* prophage by PHASTER [43]. These prophages are predicted to be “intact,” “incomplete,” or “questionable”. The “incomplete” or “questionable” described the CDSs related to the prophage gene cluster, but they did not correctly define the prophage. Most *L. curvatus* strains carry at least one intact prophage. Of note, some phage gene clusters, for example, those in *L. curvatus* strains TMW 1.167, TMW 1.1381, and TMW 1.439 [27], encode lysin. Moreover, two copies of these gene clusters are present in *L. curvatus* strains TMW 1.595, TMW 1.1390, and TMW 1.624 [27]. Further research identified all of these phages encoding lysins as “intact” or “questionable” and showed that they are encoded on chromosomes.

CRISPR combines with Cas to form a CRISPR-Cas system that provides adaptive immunity against invading components in bacteria [44]. Eisenbach et al. [27] identified nine different CRISPR-Cas groups in *L. curvatus* and found that the presence of the CRISPR-Cas system is not correlated with the source of the strain. These clusters are affected by DNA contact between the phage and other bacteria. At present, there is little research published on the CRISPR-Cas system in *L. curvatus*. The activity of the CRISPR system and its relationship with species evolution have not been studied.

Meat products are rich in arginine, which is often catabolized by arginine diimidase (ADI) pathway. Studies have shown that the *L. sakei* can degrade arginine with ammonia and ATP production by the ADI pathway, which is the key to the effective survival of this strain in meat products [36,45]. Thus, the ADI pathway is a likely energy source and a mechanism for survival in acidic environments. However, unlike *L. sakei*, an analysis of the *L. curvatus* genome has shown that *L. curvatus* does not have an ADI pathway. In fact, this is the main criterion used to distinguish *L. curvatus* from *L. sakei* [46]. An analysis of the *L. curvatus* genome showed that all strains can convert serine into pyruvic acid and NH_3_ through serine deaminase and guanine into xanthine and NH_3_ through guanine deaminase. These enzymes are not encoded in the genome of *L. sakei* 23K [36]. The generation of NH_3_ leads to a change in pH and thus enables *L. curvatus* to be acid resistant in the absence of an ADI pathway. 

## 3. Physiological and Biochemical Properties of *Latilactobacillus curvatus*

### 3.1. Carbohydrate Utilization

**Figure 2:**
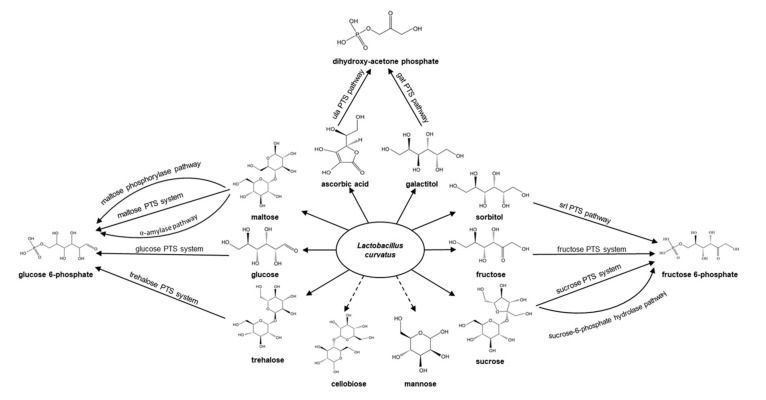
Carbohydrate utilization map of Latilactobacillus curvatus. Carbohydrates are divided into sugars and polyols. Solid lines represent carbohydrates that have evidence supporting their use by L. curvatus, and dashed lines represent carbohydrates that L. curvatus may use, but for which there is no supporting evidence. PTS: phosphotransferase.

The ability to metabolize carbohydrates is an important indicator for the cultivation and selection of bacteria. Teran et al. [26] analyzed the L. curvatus genome and found multiple carbohydrate uptake systems (Figure 2). They found that L. curvatus has at least three pathways to utilize maltose. In two of these three pathways, maltodextrins and starch are metabolized using the maltose phosphorylase and intracellular α-amylase pathways, respectively. Moreover, these two metabolic pathways are both associated with an ATP-binding cassette (ABC) transporter. A third mechanism for maltose utilization, the maltose phosphotransferase system, has been found in L. curvatus strains NRIC0822 and MRS6. This system is coupled with the malA gene, which encodes 6-phospho-α-glucosidase. Furthermore, all L. curvatus strains can also utilize glucose through the phosphotransferase system encoded by the manXYZ gene cluster. A fructose phosphotransferase system has also been found in L. curvatus, and this enables the utilization of fructose. Another phosphotransferase system related to fructose utilization has been identified in L. curvatus strains RI-406 and FLEC03. Specifically, the frl gene cluster, encoding a fructose-lysine deglycation pathway, has been detected in these strains [47]. L. curvatus strains CRL705, DSM20019, and Wikim38 contain genes encoding a trehalose phosphotransferase system, which enables them to use the α-glucan-derived disaccharide trehalose. In addition, these strains can use sucrose through two different pathways: a sucrose-6-phosphate hydrolase pathway and a pathway that involves a sucrose phosphotransferase system. Moreover, the rbsUDKR gene cluster, which is involved in ribose catabolism, has also been found in these strains. This gene cluster, which is similar to the gene cluster in L. sakei strains, encodes the ribose transporter rbsU, a protein similar to GltA, the glucose transporter of Staphylococcus xylosus [48]. Experiments by Kask et al. [6] showed that L. curvatus SSR4 and SSR6 could utilize the above six carbohydrates. In addition, these two strains can also utilize lactose, galactose, cellobiose and esculine.

Plant oils are rich in polyols, and a variety of phosphate-transfer enzyme systems specific for these compounds have also been identified in L. curvatus. L. curvatus strains, such as Wikim38 and DSM20019, contain the ula phosphotransferase pathway for ascorbic acid catabolism, which enables them to catabolize ascorbic acid [49]. Concurrently, they can utilize sorbitol and glucosyl/galactosyl alcohol through the srl and gat phosphotransferase pathways.

### 3.2. Antibiotic Resistance

Many LABs have a high antibiotic susceptibility, which is attributed to intrinsic and nontransmissible characteristics [50]. LABs are generally sensitive to cell wall inhibitors, such as penicillin (ampicillin and piperacillin) and β-lactamase inhibitors [51]. Both L. curvatus DN317 [13] and PA40 [52] show sensitivity to penicillin and ampicillin, but L. curvatus A61 [53] is resistant to ampicillin. The main mechanism of drug resistance seems to be the impermeability of cell walls, which may be the reason for the differences between strains [54]. The differences between strains may also depend on the cooperation of non-specific mechanisms, such as multidrug transporters [55] and defective cell wall autolytic systems.

Furthermore, LABs are usually sensitive to antibiotics that inhibit protein synthesis (erythromycin, tetracycline, clindamycin, and chloramphenicol), but resistant to aminoglycoside drugs (neomycin, kanamycin, streptomycin, and gentamicin) [56]. However, conflicting results have been obtained for L. curvatus strains DN317 [13] and PA40 [52]. L. curvatus DN317 [13] is resistant to chloramphenicol and sensitive to gentamicin and streptomycin, whereas L. curvatus PA40 [52] is moderately resistant to erythromycin and tetracycline. We suspect that the differences in the resistance of L. curvatus to these bacteriocins may be due to differences in resistance genes between the strains. However, there are no published data regarding the resistance genes of L. curvatus. This gap should be addressed in future studies.

In order to identify antimicrobial resistance gene in this species, complete genomes of L. curvatus MARS6 (CP022474), L. curvatus SRCM103465 (CP035110.1) and L. curvatus 20,019 (CP026116.1) were downloaded from NCBI as references and were searched using publicly available database Comprehensive Antibiotic Resistance Database (CARD). No hits for AMR genes in those three genomes were identified with the perfect/strict option for CARD [57,58]. However, under a less stringent criterion (perfect/strict/loose option), 186, 185, 167 hits in L. curvatus SRCM103465, L. curvatus MARS6 and L. curvatus 20,019 genomes were found, respectively. Fluoroquinolone, macrolide, rifamycin and elfamycin antibiotic genes with high identify (>60%) were found both the three genomes, which were considered as intraspecific features and intrinsic of L. curvatus species. Besides, the sequence of 5 kbp upstream and downstream of those genes were analyzed and no mobile elements (prophage, transposases and insertion sequences) were found, which suggested a low risk of gene transfer.

### 3.3. Auto-Aggregation and Co-Aggregation Capacity

It is generally believed that the ability of LAB to form cellular aggregates through self-aggregation (auto-aggregation) or through aggregation between genetically distinct cells (co-aggregation) is a desirable characteristic [59]. Auto-aggregation is a prerequisite for probiotics to settle in the gastrointestinal tract by which they can play a probiotic role. Additionally, probiotics co aggregation is pervasive in several ecological niches, especially in the human gut, where it may interfere with the ability of a pathogenic species to infect the host and may prevent the colonization of foodborne or non-foodborne pathogens [60]. Research by Zommiti [13] and Ahmadova [53] et al. showed that L. curvatus strains DN317 and A61 have higher auto-aggregation abilities than other strains, with auto-aggregation rates exceeding 70%. L. curvatus DN317 also showed higher adhesion ability than other strains. Its adhesion rate to Caco-2 cells reached 16%, which is almost double that of Latilactobacillus rhamnosus GG (LGG) [13]. In most cases, the aggregation ability of bacteria is related to their cell adhesion characteristics [59] and hydrophobicity is considered to be the decisive factor influencing cell adhesion [58]. However, the relationship between auto-aggregation ability, cell adhesion, and hydrophobicity of L. curvatus has not been studied.

The ability of L. curvatus to co-aggregate with pathogenic bacteria is a desirable characteristic. L. curvatus DN317 shows different co-aggregation abilities with different pathogenic bacteria, with the highest co-aggregation ability with L. monocytogenes ATCC7644 (68%) and the lowest co-aggregation ability with Campylobacter jejuni National Collection of Type Cultures (NCTC) 11168 (35%) [13]. Furthermore, L. curvatus A61 shows different co-aggregation abilities with different strains of the same pathogen [53]. This indicates that the co-aggregation ability of L. curvatus with pathogenic bacteria differs by the species and strains of the pathogenic bacteria. 

### 3.4. Resistance to Gastrointestinal Tract Conditions

Resistance to the extreme environment of the gastrointestinal tract is an important characteristic used for the evaluation of bacterial strains. The mouth is the first barrier that must be overcome because saliva contains a high concentration of lysozyme. The next barrier is the stomach, because it maintains a low pH and harbors digestive enzymes. The final barrier is the upper intestine, which contains bile [61,62]. Zommiti et al. [13] treated L. curvatus DN317 with 100 mg/L lysozyme for 30 and 120 min and found that its survival rate was higher than 70%. The resistance of the strain to lysozyme is mainly due to the peptidoglycan structure of the cell wall, the physiological state of the cell, and the lysozyme concentration in the culture medium [63].

Most microorganisms are inactivated by the strong acidic conditions in the stomach [64]. Probiotics must survive the low pH of the stomach to perform their various physiological functions [65]. L. curvatus PA40, which was isolated by Hong et al. [52], shows a high survival rate of 97.8% in 1% pepsin at pH 2.5. Zommiti et al. [13] found a similar result with L. curvatus DN317, which remains viable at pH 2.5. This indicates that L. curvatus may be able to withstand the low pH conditions of the stomach. L. curvatus may prevent the entry of H^+^ by changing the structure and permeability of the cell membrane [66] or the exopolysaccharides produced by it can provide it with the ability to tolerate an acidic environment [67]. It may also produce NH_3_ to change the pH of the environment. However, this is only a hypothesis, which needs some research to support it.

Bile acid resistance is also an important characteristic used to evaluate bacterial strains. Mathara et al. [68] determined a limit of 0.3% bile for strain selection, and reported that suitable resistance to bile is indicated by a growth percentage higher than 50% in the presence of 0.3% bile. Erkkilä and Petäjä [69] measured the bile acid tolerance of L. curvatus strains and found that strains derived from commercial meat starter cultures were resistant to 0.3% bile salt at pH 6. Similarly, Ahmadova et al. [53] observed the growth of L. curvatus A61 at bile acid concentrations of 0.2% and 0.3%. At present, the mechanism of bile resistance of L.curvatus is not clear. The efflux of bile salts, the hydrolysis of bile salts [70] and the production of exopolysaccharides and other common mechanisms of resistance to bile salts by lactic acid bacteria can be an entry point for future research on the mechanisms of bile resistance of L. curvatus.

### 3.5. Generation and Degradation of Biogenic Amines

Biogenic amines are low-molecular weight nitrogen-containing compounds of biological importance [71]. They are mainly formed by amino acid decarboxylation [72] and exist in most fermented foods, such as sausage, wine, cheese, yoghurt, and beer [73,74,75]. In fermentation culture, L. curvatus can hydrolyze proteins to release free amino acids, which are further decarboxylated to produce biogenic amines. In recent years, many studies have shown that L. curvatus has genes encoding tyrosine decarboxylase and ornithine decarboxylase, and thus, it can generate tyramine and putrescine [76,77]. In addition, due to the similar structure of phenylalanine and tyrosine, tyrosine decarboxylase can decarboxylate phenylalanine to produce β-phenylethylamine [76,78]. L. curvatus does not contain a gene encoding lysine decarboxylase but has low cadaverine-forming ability, which may be the result of ornithine decarboxylase activity against lysine, because lysine and ornithine have similar chemical structures [79,80]. Further studies have found that L. curvatus does not contain a gene encoding histidine decarboxylase and thus, has no potential to produce histamine [81].

In the first stage of sausage fermentation, tyramine is the main biogenic amine produced by L. curvatus. In contrast, the production of putrescine occurs at a later stage and at a slower rate than tyramine, yielding lower final levels. Phenylethylamine accumulates at much lower levels than tyramine and putrescine. Its accumulation usually begins in the second half of the maturation process and is accompanied by the production of large quantities of tyramine [82].

LAB not only produce biogenic amines through the decarboxylation of amino acids, but also synthesize amine oxidase to degrade biogenic amines [83,84]. Li et al. [76] found that L. curvatus may possess a multi-copper oxidase that degrades biogenic amines. They also screened a strain with high biological amine degradation ability and low biological amine production ability, namely L. curvatus G-1, a promising candidate for the control of biogenic amine levels in fermented meat products.

### 3.6. Production of Bacteriocin

Bacteriocins, which are produced by LAB, are peptides synthesized by ribosomes or small proteins secreted into the environment. Their production is the main strategy used by microorganisms to survive and compete for limited space and nutrients in their ecosystem [85]. They usually act on closely related microorganisms and some Gram-positive pathogens associated with food spoilage and diseases [86]. Based on their physicochemical properties, bacteriocins have been divided into two main categories: lanthionine-containing lantibiotics (class I) and nonlanthionine-containing bacteriocins (class II) [87].

In recent years, some bacteriocins produced by L. curvatus have been purified and characterized. These include curvacin A, produced by L. curvatus LTH1174 isolated from fermented sausages [88], sakacin G produced by L. curvatus ACU-1 isolated from artisanal dry fermented sausages [89], sakacin P and sakacin X, produced by L. curvatus MBSa2 and MBSa3 isolated from Italian salami [21], curvaticin 13, produced by L. curvatus SB13 isolated from semidry sausages [90], lactocin AL705, produced by L. curvatus CRL705 isolated from fermented sausages [91] and curvaticin 422, produced by L. curvatus L422 isolated from fermented sausages [92] (Table 2). These are all class II bacteriocins. Their production occurs in the exponential growth phase, during which their activities continue to increase. Moreover, they can tolerate a wide range of pH and temperature conditions [53,93], and have antibacterial activities against a wide spectrum of pathogenic and spoilage bacteria, such as Bacillus cereus, L. monocytogenes, S. aureus and Enterococcus faecium [21,94,95,96]. Their stability and broad antibacterial spectrum make them potential bioprotective agents in the fermentation of meat products [21].

Among the bacteriocins produced by L. curvatus reported thus far, sakacin G and curvacin A have been studied most extensively. Curvacin A was the first bacteriocin identified and characterized from a strain of L. curvatus LTH1174 [22]. Amino acid composition analysis and automated protein sequencing revealed that it is a small peptide of 38–41 amino acid residues. Curvacin A does not contain unusual amino acids, such as lanthionine, but contains an N-terminal alanine. It is degraded by proteinase K and trypsin, but not by pepsin, bovine serum albumin, or RNase. Curvacin A inhibits the growth of the food pathogens L. monocytogenes and E. faecalis and thus can enhance the performance of starter cultures and improve the condition of meat products [102].

In 2002, Simon et al. [103] were the first to isolate sakacin G from L. sakei 2512. It was later identified in L. curvatus ACU-1. Sakacin G is a 37-amino acid class IIa bacteriocin encoded by the duplicated structural genes skgA1 and skgA2. It has two disulfide bonds, of which one (C-terminal) is necessary for antibacterial activity. Due to the existence of double-disulfide bridges that are vital for its antimicrobial activity, sakacin G is unique and is known as an intermediate between pediocin-like bacteriocins. Moreover, it belongs to the mesentericin-like bacteriocins, based on sequence homology and its inhibition spectrum and specific activity. Sakacin G has strong anti-Listeria activity, which make it suitable for use as an antibacterial peptide to reduce or eliminate the growth of pathogenic bacteria and improve the quality, safety, and shelf life of food. In addition, the sakacin G promoter can be used as a tool to induce a high-level expression of other bacteriocins [89].

## 4. Applications of *Latilactobacillus curvatus* in Fermented Meat Products and Food Packaging

*L. curvatus* has a remarkable ability to produce bacteriocins with strong anti-Listeria activity and the ability to inhibit some spoilage bacteria in meat products [21]. As a native bacterium in meat products, it also plays a certain role in the maturation of these products and the formation of desirable flavor [30]. Therefore, *L. curvatus* is often used in the food industry as a starter for fermented sausages [104] and as a biological protection culture for meat products [30]. In addition, it can be used to produce active food-packaging films [28].

### 4.1. Starter for Meat Products

Meat fermentation is a preservation technology with a long history. LAB, especially L. plantarum, *L. curvatus*, *Pediococcus acidilactici*, *L. sakei*, and *P. pentosaceus*, are involved in the processing of all types of fermented sausages. In Europe, fermented sausages are manufactured using starter cultures containing mainly *L. sakei* and *L. curvatus* [105]. Recently, many studies have found that some bacteriocin-producing *L. curvatus* strains, such as LTH1174 [106], 54M16 [107], MBSa2 and MBSa3 [21], can reduce the number of *L. monocytogenes*, a major problem in fermented sausages [108], by a greater degree than can a nonbacteriocinogenic control culture. Zhang et al. [109] found that *L. curvatus* can inhibit the growth of the spoilage bacteria, *Enterobacteriaceae*, *Pseudomonas fragi* and *Brochothrix thermosphacta*, which are common in meat products during storage. It can also inhibit the growth of *Pseudomonas putida* at the later stage of storage. *L. curvatus* significantly reduces microbial diversity in meat products and inoculated samples. Ripening has been shown to be almost completely carried out by *Latilactobacillus*. This indicates that bacteriocin produced by *L. curvatus* can control the fermentation process by inhibiting the growth of specific bacteria and competitive microbial communities and thus improve the safety of food products [104]. Further research by Stella et al. [110] showed that the inhibitory properties of LAB such as *L. curvatus*, are closely related to the production of acids (e.g., lactic and acetic acids), bacteriocins and hydrogen peroxide, and the competition of two strains for limited nutrients.

Another potential safety hazard during sausage fermentation is sodium nitrite. Sodium nitrite is a key component in the curing process of meat products. It can promote the formation of color and flavor in meat products and inhibit the growth of harmful bacteria [111,112]. However, when it reacts with secondary amines, it produces amine nitrite, a carcinogenic, teratogenic, and mutagenic compound [113]. Sun et al. [29] found that *L. curvatus* has a strong inhibitory effect against four types of amine nitrites detected in Harbin dry sausage. *L. curvatus* can decompose nitrosamines and fatty acids through a series of specific enzyme systems. However, it also has a high acid-production capacity, which can reduce the pH of the fermentation system and subsequently cause the reduction of nitrite, nitrite residues, and N-nitrosamines [114,115,116].

As a starter culture, *L. curvatus* can not only improve the safety of fermented sausage, but also promote the formation of its flavor. Casaburi et al. [101] found that *L. curvatus* 54M16 was able to hydrolyze sarcoplasmic protein to produce peptides and free amino acids. During the ripening of sausage, these peptides and free amino acids can be used directly as flavor and taste enhancers or as substrates for many meat microorganisms, to produce various aromatic compounds [117]. Furthermore, *L. curvatus* 54M16 also has the ability to promote the release of short chain fatty acids. Under the environmental conditions used to produce dry fermented sausage, the ability of a meat starter culture to hydrolyze esters and release short-chain and medium-chain free fatty acids may affect the taste and flavor of the sausage.

### 4.2. Food Packaging

Antimicrobial packaging systems are part of an emerging technology designed to control the number of microorganisms and inhibit the growth of specific microorganisms, thereby increasing the safety and quality of food products. Various chemical preservatives have been used in active antimicrobial-releasing systems. Among these preservatives, bacteriocins are most commonly incorporated into films [118]. Bacteriocins derived from *L. curvatus* has been widely used in active food packaging. Mauriello et al. [28] and Massani et al. [119] sprayed bacteriocin solutions produced by *L. curvatus* strains 32Y and CRL705 on polyethylene films to produce active food-packaging films. After a period of time, the films showed stable anti-Listeria activity, and heat treatment at 70 °C did not affect the antibacterial activity of the films. Massani et al. [119] further compared an active polyethylene film adsorbed with lactocin 705 and AL705 produced by *L. curvatus* CRL705 with a polyethylene film incorporated with nisin, which is the most commonly used antibacterial agent [118]. Compared with nisin-treated film, the lactocin-treated active polyethylene film was shown to inhibit Listeria more effectively, and the functional properties of the film were not affected. Massani et al. then studied the factors affecting the adsorption of *L. curvatus* bacteriocin on to the polyethylene film [120]. Temperature and time were found to affect bacteriocin adsorption on to the polyethylene film to some extent, with 60 min and 30 °C identified as the best conditions for adsorption. Impurities produced by the growth of *L. curvatus* strongly affect the adsorption and antibacterial activity of bacteriocins on polyethylene film. Bacteriocin activity decreases with increasing impurity concentration. This may be caused by competitive adsorption between bacteriocins and impurity molecules, which provides an idea for improving the antibacterial activity of food packaging film.

## 5. Probiotic Function of *Latilactobacillus curvatus*

Since 2013, researchers have studied the effects of *L. curvatus* on human health. Although this research is still in the initial stages, results show that *L. curvatus* can relieve obesity and hyperlipidemia, but it was more effective when mixed with *L. plantarum* [31,32] and this species can also relieve the symptoms of colitis in mice [33,34]. Recent research shows that *L. curvatus* can also effectively prevent muscle atrophy induced by dexamethasone [35].

### 5.1. Obesity

Obesity is defined by the World Health Organization as abnormal or excessive fat accumulation that can damage health and is considered a global epidemic. It is a typical metabolic syndrome disease that is closely associated with type two diabetes, hypertension, dyslipidemia, and nonalcoholic fatty liver disease [121]. Recent studies have shown that the human gut microbiota may have a critical impact on the onset and development of obesity [122]. Thus, the modification of the gut microbiota has become an important method to treat obesity [123,124]. Probiotics can actively regulate the host gut microbiota to improve metabolic disorders, which is an important asset for the treatment of obesity [125].

In 2013, Yoo et al. [34] were the first to show that a combination of *L. curvatus* and *Latilactobacillus plantarum* can be used to treat diet-induced obesity. They used probiotics (*L. curvatus* HY7601 and *L. plantarum* KY1032) to treat mice with diet-induced obesity for 10 weeks and found that body weight and gut microbiota diversity both decreased after probiotic treatment. In the gut, probiotics can change the composition of gut microbes in many ways. They can reduce the diversity of gut microbiota by competing with certain nutrients or secreting antibacterial proteins [32]. Recent studies have also shown that low diversity of the gut microbiota has a certain protective effect against diet-induced obesity in mice [126]. However, there is controversy about the effect of probiotic supplementation on gut microbiota and whether there are any long-term consequences of prolonging probiotic supplementation, which need to be further studied. In addition, Jeung et al. [127] found that the ratio of Firmicutes to Bacteroidetes in the gut of mice increases after treatment, which may be due to the increased abundance of *Lachnospiraceae*, which belong to the phylum Firmicutes. An increase in the abundance of *Lachnospiraceae* can result in a greater production of butyrate, thus preventing colon cancer and fat accumulation [128].

Probiotics can not only treat obesity by regulating the gut microbiota, but also play a role in inhibiting adipocyte differentiation and reducing fat accumulation [129]. Shim et al. [130] found that the mixture of *L. curvatus* HY7601 and L. plantarum KY1032 decreased adipogenesis in 3T3-L1 cells by regulating the main transcription factors related to adipogenesis and by reducing mitochondrial biogenesis which inhibits the ATP supply required for adipocyte differentiation. In HepG2 cells, the mixture of two *Latilactobacillus* spp. decreases the expression of SREBP-1c, thus inhibiting the central enzyme in the de novo lipogenesis pathway, fatty acid synthetase and acetyl-CoA carboxylase. Jung et al. [131] found that the weight loss induced by *L. curvatus* HY7601 was related to a reduction in fat mass, which was associated with changes in Lp-PLA2 activity. Supplementation with *L. curvatus* reduced Lp-PLA2 activity and oxidized low-density lipoproteins (LDL), increasing the particle size of LDLs and thus reducing fat accumulation.

### 5.2. Dyslipidemia

Dyslipidemia is characterized by an increase in the blood levels of total or LDL-cholesterol and triglycerides, or a decrease in high-density lipoprotein-cholesterol levels. It is a risk factor for cardiovascular disease [132]. Dyslipidemia can be divided into hypercholesterolemia, hypertriglyceridemia and mixed hyperlipidemia. In the past decade, probiotics have rapidly emerged as a natural therapy with the potential to improve dyslipidemia [133]. Ahn et al. [33,134] first discovered a combination of *L. curvatus* and *L. plantarum* can reduce triglyceride levels in patients with hypertriglyceridemia in 2015. The consumption of *L. curvatus* HY7601 and *L. plantarum* KY1032 for 12 weeks has been shown to reduce triglyceride levels and increase the particle size of apo A-V and LDL in hypertriglyceridemic subjects. Apo A-V is implicated in triglyceride metabolism and may be a potent factor affecting plasma triglyceride levels in humans. Apo A-V can accelerate the hydrolysis of triglycerides in plasma, by enhancing the activity of LPL, thus reducing plasma triglyceride levels [135]. Choi et al. [136] reported a similar result, that is, *L. curvatus* HY7601 and L. plantarum KY1032 lower triglycerides in hypertriglyceridemic rats by upregulating ApoA-V, PPARα, and FXR. *L. curvatus* can also improve hypercholesterolemia to some extent. Park et al. [137] demonstrated that dietary supplementation with the probiotics *Leuconostoc mesenteroides* subsp. *mesenteroides* KDK411 and *L. curvatus* KFP419, isolated from kimchi, is effective at lowering blood cholesterol levels and alleviating hypercholesterolemia in rats by increasing fecal excretion of cholesterol and coprostanol through cholesterol assimilation by the bacteria.

### 5.3. Others

Dextran sodium sulfate (DSS)-induced colitis shorten the colon of mice and lead to the destruction of colon mucosal epithelia, severe infiltration of inflammatory cells, and edematous lesions in the submucosa layer. Jo et al. [31] found that the administration of *L. curvatus* Wikim38 effectively alleviates these symptoms in mice. The mechanism for this effect may be that *L. curvatus* mediates the production of IL-10 in dendritic cells through NF-κB and extracellular regulated protein kinases (ERK) signaling. This is consistent with the mechanism used by *L. brevis* to alleviate trinitrobenzene sulfonic acid-induced colitis in mice.

Recently, Katsuki et al. [35] found that *L. curvatus* CP2998 prevents dexamethasone-induced muscle atrophy of C2C12 skeletal muscle cells. This is the first report of the inhibition of muscle atrophy by LAB. After treatment with *L. curvatus* CP2998, the diameter of the myotubes increased and the mRNA expression levels of MuRF1, MAFbx, and E3 ubiquitin ligase decreased. Meanwhile, *L. curvatus* CP2998 was also found to inhibit glucocorticoid-dependent transcription. In brief, *L. curvatus* prevents glucocorticoid-induced muscle atrophy by inhibiting the activation of the glucocorticoid receptor. This indicates that *L. curvatus* CP2998 may have a new application in the treatment of muscle atrophy.

## 6. Conclusions and Future Perspectives

As a candidate probiotic, *Latilactobacillus curvatus* shown to have a variety of genes associated with carbohydrate utilization and bacteriocin producing, which may enable it strong carbohydrate fermentative ability and antibacterial ability. Besides, this species has suitable auto-aggregation and co-aggregation abilities, which enable it to colonize the intestinal tract and effectively eliminate pathogens. Due to these genomic and physiological characteristics, *L. curvatus* has great application potential in the food industry and in promoting human health. However, it is worth noting that some *L. curvatus* are also producers of bioamines, which may be a threat to human health. This is a point that cannot be ignored in its applications.

Currently, research on the *L. curvatus* genome and population-based genetic analyses of *L. curvatus* in large sample sets are lacking. Moreover, data regarding the evolution, genetic characteristics, and host effects of *L. curvatus* are not available. Therefore, it is necessary to study the evolutionary model of *L. curvatus*, based on population genomics, and to link this evolutionary model with the metabolism, function, and phylogeny of the strain, to lay a foundation for research on the probiotic function of this species. *L. curvatus* strains with different probiotic functions may also be suitable for use in the development of functional fermented products to broaden their application in the food industry. In addition, the probiotic function of *L. curvatus* has not been demonstrated clinically. To address the gaps in our understanding of the role of *L. curvatus* in immune regulation and to use it clinically, further research is needed to clarify the mechanism of *L. curvatus* in disease treatment.

## Figures and Tables

**Table 1:** Genome Sequences of *Latilactobacillus curvatus* Strains.

Strain	Source	Genome Size (Mb)	GC Content (%)	Number of CDS	Accession Number	Sequencing Status (Sequencing Technology)	Reference
*L. curvatus* FBA2	Fermented vegetables	1.849	42.10	1711	CP016028	Complete (PacBio RS II platform)	[8]
*L. curvatus* Wikim38	Kimchi	1.940	41.93	1885	CP017124	Complete (PacBio RS II platform)	[37]
*L. curvatus* Wikim52	Kimchi	1.987	42.00	1875	CP016602	Complete (PacBio RS II platform)	NP
*L. curvatus* KG6	Meat	2.002	42.03	1970	CP022475	Complete (PacBio RS II platform)	[40]
*L. curvatus* MRS6	Meat	2.114	41.70	1975	CP022474	Complete (PacBio RS II platform)	[40]
*L. curvatus* NFH-Km12	Traditional Japanese fermented fish	1.989	41.81	1946	AP018699	Illumina MiSeq pair-end	[39]
*L. curvatus* TMW 1.421	Sausage	1.994	41.97	1961	CP016221	Complete (PacBio RS II platform)	[27]
*L. curvatus* TMW 1.439	Sausage	1.948	42.04	1939	CP015489	Complete (PacBio RS II platform)	[27]
*L. curvatus* TMW 1.624	Sausage	2.132	41.63	2148	CP015490	Complete (PacBio RS II platform)	[27]
*L. curvatus* TMW 1.595	Starter culture	2.032	41.95	1991	CP016470	Complete (PacBio RS II platform)	[27]
*L. curvatus* TMW 1.1381	Starter culture	1.949	42.05	1993	CP015493	Complete (PacBio RS II platform)	[27]
*L. curvatus* TMW 1.1390	Starter culture	1.977	42.07	1949	CP015494	Complete (PacBio RS II platform)	[27]
*L. curvatus* TMW 1.401	Sauerkraut	1.886	42.00	1830	CP016216	Complete (PacBio RS II platform)	[27]
*L. curvatus* TMW 1.407	Sauerkraut	1.886	42.01	1831	CP016218	Complete (PacBio RS II platform)	[27]
*L. curvatus* TMW 1.27	Unknown	2.056	41.86	2027	CP016467	Complete (PacBio RS II platform)	[27]
*L. curvatus* TMW 1.167	Unknown	1.951	42.03	1940	CP016472	Complete (PacBio RS II platform)	[27]
*L. curvatus* FLEC03	Beef	1.902	41.70	1926	GCA_900178545.1	Draft (Illumina MiSeq pair-end)	[38]
*L. curvatus* RI-124	Meat	1.810	42.00	1838	MKDR00000000	Draft (Illumina MiSeq pair-end)	[41]
*L. curvatus* RI-193	Meat	1.805	42.00	1862	MKGD00000000	Draft (Illumina MiSeq pair-end)	[41]
*L. curvatus* RI-198	Meat	1.804	42.00	1848	MKGC00000000	Draft (Illumina MiSeq pair-end)	[41]
*L. curvatus* RI-406	Meat	2.001	41.70	2020	MKDG00000000	Draft (Illumina MiSeq pair-end)	[41]
*L. curvatus* CRL705	Argentinean fermented sausages	1.838	41.90	1830	AGBU01000000	Draft (454 GS Titanium pyrosequencing)	[25]
*L. curvatus* NRIC0822	Kabura-zushi	1.945	41.80	1831	GCA_000805355.1	Draft (Illumina HiSeq pair-end)	[42]
*L. curvatus* DSM20019	Milk	1.917	41.99	1828	GCA_004101845.1	Draft (Ion Torrent PGM)	NP

NP, no publication available.

**Table 2:** Bacteriocins Produced by Latilactobacillus curvatus.

Bacteriocin-Producing Strain	Bacteriocin	Source	Active Against	Reference
*L. curvatus* LTH1174	Curvacin A	Fermented sausages	*Enterococcus faecalis*, *Listeriamonocytogenes*	[88]
*L. curvatus* SB13	Curvaticin 13	Semidry sausages	*L. monocytogenes*, *Staphylococcus aureus*	[90]
*L. curvatus* FS47	Curvaticin FS47	Beef	*L. monocytogenes*	[97]
*L. curvatus* L422	Curvaticin L422	Fermented sausages	*L. monocytogenes*	[92]
*L. curvatus* CRL705	Lactocin 705	Argentine fermented sausage	*L. monocytogenes*	[91]
*L. curvatus* DN317	Curvaticin DN317	Chicken Ceca	*Campylobacter jejuni*, *L. monocytogenes*, *Bacillus subtilis*	[98]
*L. curvatus* 54M16	Sakacin X, P, T	Fermented sausages	*Staphylococci*, *Enterobacteriaceae*	[99]
*L. curvatus* A61	Curvacin A	Azerbaijani cheese	*L. monocytogenes*, *B. cereus*	[53]
*L. curvatus* BCS35	SakacinP-H12Y, Sakacin X	Dry-salted cod	*L. monocytogenes*	[100]
*L. curvatus* ACU-1	Sakacin G, P, Q	Argentine fermented sausage	*L. monocytogenes*	[89]
*L. curvatus* MBSa2	Sakacin P, X	Salami	*L. monocytogenes*	[21]
*L. curvatus* CWBI-B28	Sakacin P	Raw poultry meat	*L. monocytogenes*	[101]
